# Book Reviews

**Published:** 1913-05

**Authors:** 


### BOOK REVIEWS

New Towns and Business Opportunities. Booklet sent free upon request by the Chicago, Milwaukee & St. Paul Ry., Chicago.

In its sixty-four pages are listed the population, resources and general characteristics of 250 towns and small cities of recent origin where the growth in population has exceeded the development of the needed businesses and professions.

The first list contained in the booklet is that of the businesses and professional openings which the towns offer. The businesses and professions are listed alphabetically, and each is followed by the names of the towns and cities which now need such a business or professional service as is named.

Then follow two lists of names of towns, each giving briefly such information as men and women need in determining the desirability of the community for their occupations. One of these lists is made up of towns and small cities along the eastern lines of the railroad, the other being made up of communities along the Puget Sound lines. The booklet also contains a comprehensive map showing the location of the towns and the railroad connections.

Golden Rules of Gynecology. By George B. Norberg, M. D., Professor of Diseases of Women and Clinical Gynecology, University Medical College, Kansas City, Mo., Gynecologist to Kansas City General Hospital, Fellow and ex-President Kansas City Academy of Medicine; 250 pages; 8 vo.; price, $2.25. C. V. Mosby Co., St. Louis.

There is a need for just such a book as this one. It does not displace the textbook or the monograph on gynecology, but is rather a guide to what one should know and observe on this fascinating branch of medicine.

In 250 pages one finds the really "Golden Rules"—the observance and application of which will enable the practitioner of medicine to get results. Convenient in size and convincingly written, this volume can be perused over and over again with the feeling that each time it is read one becomes better able to cope with diseases of women.

---

Tuberculin in Diagnosis and Treatment, by Francis Marion Pottenger, A. M., M. D., LL. D., Medical Director of the Pottenger Sanatorium for Diseases of the Lungs and Throat, Monrovia, California. The C. V. Mosby Company, Medical Publishers, 801-806 Metropolitan Bldg., St. Louis. 243 pages, royal octavo, 35 illustrations, including one colored plate. Price, $3.00.

This volume is the most complete and up-to-date work on tuberculin that has yet appeared. Beginning with the importance of tuberculin tests in the early diagnosis of tuberculosis, the author discusses in detail "Subcutaneous Tuberculin Test," "Cutaneous Tuberculin Test," "Tuberculin in Treatment of Tuberculosis," "Hypersensitiveness," "Certain Conditions which have made the Adoption of Tuberculosin as a Diagnostic and Therapeutic Measure Difficult," "Evidences of the Therapeutic Value of Tuberculin," "Fever in the Relationship to Tuberculosis," "Temperature Curve in Tuberculosis," "Technic of Administering Tuberculin," and an Appendix, in which is given for the first time in English, Koch's announcement of the discovery of tuberculin.

Dr. Pottenger is qualified to speak on this subject. Two thousand cases of tuberculosis coming under his personal care in sanatorium practice furnishes the basis for this work. Careful painstaking effort, is everywhere noticeable in this production. The chapters on Importance of the Tuberculin Test in the Early Diagnosis of Tuberculosis is especially to be commended, as well as that on Technique on Administering Tuberculin.

There is no doubt but that many failures attending the use of tuberculin in the past have been due to a lack of knowledge of its proper administration. This defect can be overcome by a careful perusal of this volume and by following its technique.

---

Keene's Surgery, Volume VI, by 81 eminent surgeons, edited by W. W. Keen, M. D., LL. D., Hon. F. R. C. S. (Eng. and Edin.), Emeritus Professor of the Principles of Surgery and of Clinical Surgery, Jefferson Medical College, Phila. Octavo of 1177 pages, with 519 illustrations, 22 in colors. Philadelphia and London: W. B. Saunders Company, 1913. Entire work, consisting of six volumes, per volume, cloth, $7.00 net; half morocco, $8.00 net.

The original five volume work by this distinguished author and his collaborators was issued between 1906 and 1909. The present is, therefore, in the nature of a supplement, incorporating recent advances in surgical pathology and clinical surgery. While it includes a general index to all six volumes, the present volume is really an independent work, consisting of a series of elaborate monographs on modern surgery, using the word modern in its most limited sense; so that it is a valuable addition to the surgeon's library whether he has the original series or not. We are pleased to note that the first article, on Inflammation, is contributed by our Associate Editor, Dr. J. George Adami.

---

Disease in Milk: the Remedy Pasteurization. Compiled by Mrs. Lina Guthers Straus; 221 pages; illustrated.

This book is the record and the lesson of one of the greatest philanthropies ever undertaken. It contains copies of various circulars for instructing the poor and ignorant and statistics of the utmost value for social workers along similar lines. Interesting personal notes of Mr. Nathan Straus's work are appended. We do not quite agree with the implication in the title that Pasteurization is the sole remedy for milk-borne disease, in all places and under all circumstances though it certainly is the most practical at present, for large cities depending on a mixed milk supply. From 1891 to 1911, the annual death rate for children under five, in New York City, declined from 96.5 per 1000 to 41.8. The corresponding death rates for the hot season, June-August, declined from 126.4 to 44.6. Not only has Mr. Straus's philanthropy had a direct effect in improving conditions among the most vulnerable class, but it has had an even greater and far-reaching influence by example and by solving problems for similar institutions and for the country at large. In this work he has been ably seconded by his devoted wife.

With some hesitation, we take this occasion to call attention to an injustice—one of many—done to Mr. Straus's race. A book the size of this report could be compiled, consisting of stories reiterating the stinginess of the Jew. As a matter of fact, the Jew asks the least charity and is the most generous contributor to charity of any race represented in this great boarding house of the world.

---

Text Book of Physiology. Isaac Ott, A. M., M. D., Philadelphia. F. A. Davis Co., Philadelphia; 4th edition; 911 pages; 434 illustrations, many half tones and in color.

Following the usual order of text books on physiology, the author has incorporated much of the newer science, developed from clinical studies of the ductless glands, metabolism, special sense organs and the nervous system. Unfortunately, the allusions to the physiology of articulate speech show a complete lack of conception. Otherwise, the work is excellent.

